# Myocarditis or ‘hot phase’ of arrhythmogenic cardiomyopathy? A case series

**DOI:** 10.1093/ehjcr/ytaf460

**Published:** 2025-09-18

**Authors:** André Ferreira, Rita Teixeira, Pedro Brás, José Viegas, Inês Almeida, Diana Antunes, Rui Cruz Ferreira, Sílvia Aguiar Rosa

**Affiliations:** Department of Cardiology, Hospital de Santa Marta, Unidade Local de Saúde de São José, R. de Santa Marta 50, 1169-024 Lisbon, Portugal; Department of Cardiology, Hospital de Santa Marta, Unidade Local de Saúde de São José, R. de Santa Marta 50, 1169-024 Lisbon, Portugal; Department of Cardiology, Hospital de Santa Marta, Unidade Local de Saúde de São José, R. de Santa Marta 50, 1169-024 Lisbon, Portugal; Department of Cardiology, Hospital de Santa Marta, Unidade Local de Saúde de São José, R. de Santa Marta 50, 1169-024 Lisbon, Portugal; Department of Cardiology, Hospital de Santa Marta, Unidade Local de Saúde de São José, R. de Santa Marta 50, 1169-024 Lisbon, Portugal; Department of Cardiology, Hospital de Santa Marta, Unidade Local de Saúde de São José, R. de Santa Marta 50, 1169-024 Lisbon, Portugal; Department of Cardiology, Hospital de Santa Marta, Unidade Local de Saúde de São José, R. de Santa Marta 50, 1169-024 Lisbon, Portugal; Department of Cardiology, Hospital de Santa Marta, Unidade Local de Saúde de São José, R. de Santa Marta 50, 1169-024 Lisbon, Portugal

**Keywords:** Arrhythmogenic cardiomyopathy, Myocarditis, Hot phase, Genetic mutation, DSP, LMNA, Cardiac magnetic resonance, Case series

## Abstract

**Background:**

Arrhythmogenic cardiomyopathy (ACM) is a genetic condition characterized by fibrofatty replacement of myocardial tissue, leading to arrhythmias and structural heart changes. Recent studies have identified an acute inflammatory phase, or ‘hot phase’, within the progression of ACM that presents with clinical features similar to myocarditis. This phase complicates the differentiation between ACM and myocarditis, posing a diagnostic challenge.

**Case summary:**

We present two cases of young male patients, both with mutations in the DSP and LMNA genes, who initially presented with symptoms of myocardial inflammation. Patient 1, a 23-year-old male, presented with pleuritic chest pain, elevated troponin, and imaging findings suggesting myocarditis. Cardiac magnetic resonance (CMR) revealed extensive subepicardial late gadolinium enhancement (LGE) in a non-ischaemic pattern. Genetic testing confirmed a likely pathogenic (LP) LMNA mutation. Patient 2, a 26-year-old male with family history of sudden cardiac death, presented similarly with chest pain and elevated biomarkers. His CMR showed intramural LGE, and genetic testing identified a LP DSP mutation. He underwent implantation of a subcutaneous defibrillator (ICD) due to arrhythmic risk.

**Discussion:**

This case series underscores the importance of recognizing the ‘hot phase’ of ACM, which can clinically mimic myocarditis. Cardiac magnetic resonance is crucial for differentiating these entities, while genetic testing confirms the diagnosis, offering prognostic information. Mutations in the LMNA and DSP genes, particularly associated with inflammation in ACM, require consideration of arrhythmia prevention strategies, such as ICD implantation. Multidisciplinary management and advanced imaging play essential roles in the care of these patients.

Learning points

- Recognizing the ‘hot phase’ of arrhythmogenic cardiomyopathy (ACM) is essential as it mimics myocarditis, impacting diagnosis and management.
- Advanced imaging with cardiac magnetic resonance (CMR) and genetic testing are key tools for distinguishing ACM from myocarditis, guiding risk stratification and treatment options.

## Introduction

Arrhythmogenic cardiomyopathy (ACM) is characterized by the gradual replacement of myocardial tissue with fibrofatty deposits, leading to electrical and structural abnormalities within the heart. Although historically perceived as a chronic condition, recent research has identified an acute inflammatory phase in ACM progression, often referred to as the ‘hot phase’. This phase mirrors the clinical and imaging manifestations typical of myocarditis, thus rendering the differentiation between ACM in its ‘hot phase’ and myocarditis a diagnostic challenge.^1-3^ We report two cases of young male patients, one with a mutation in the DSP gene and the other with a mutation in the LMNA gene, both presenting features of myocardial inflammation as the first clinical manifestation.

## Summary figure

**Figure:**
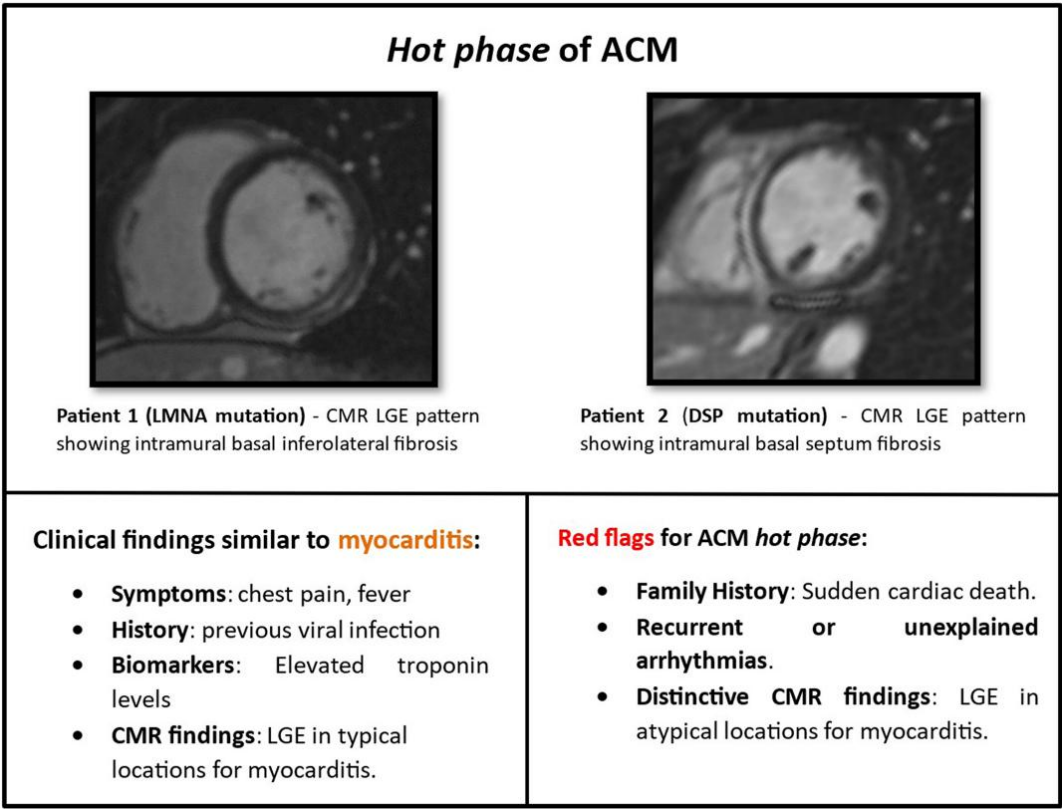


## Case presentations

### Patient 1

**Figure 1:**
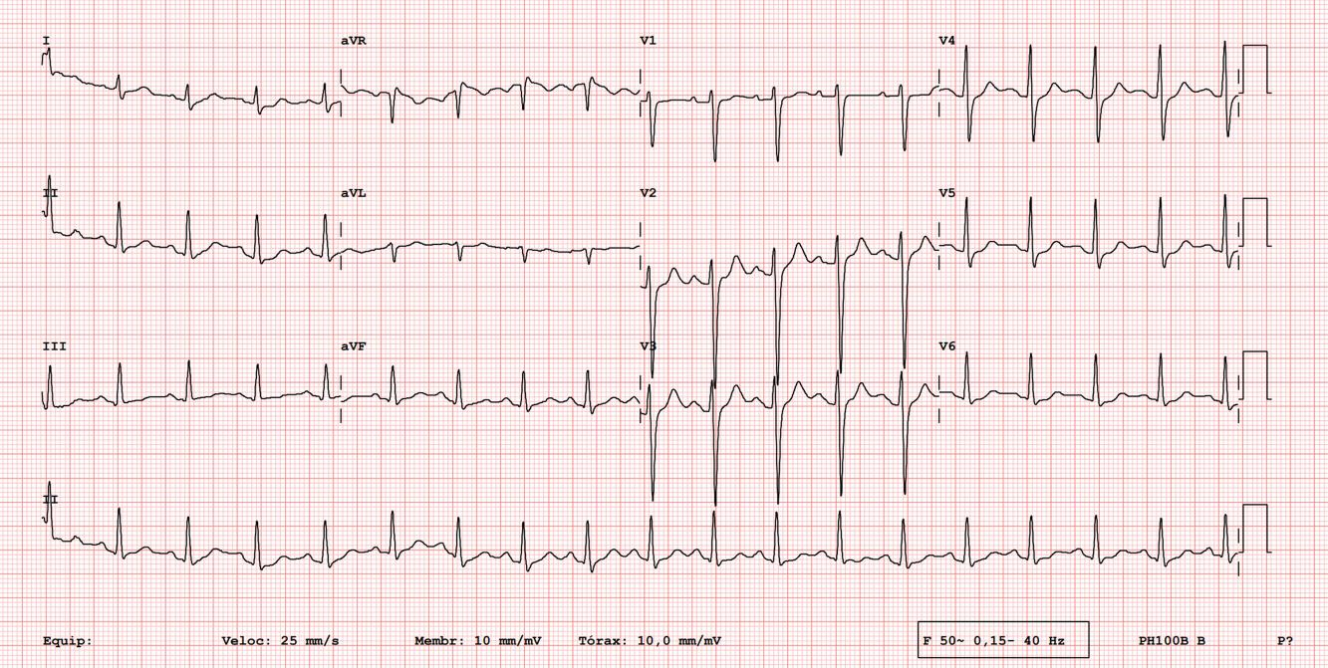
Patient 1 electrocardiogram showing sinus tachycardia, slight PR, and ST-segment depression in v3–6.

**Figure 2:**
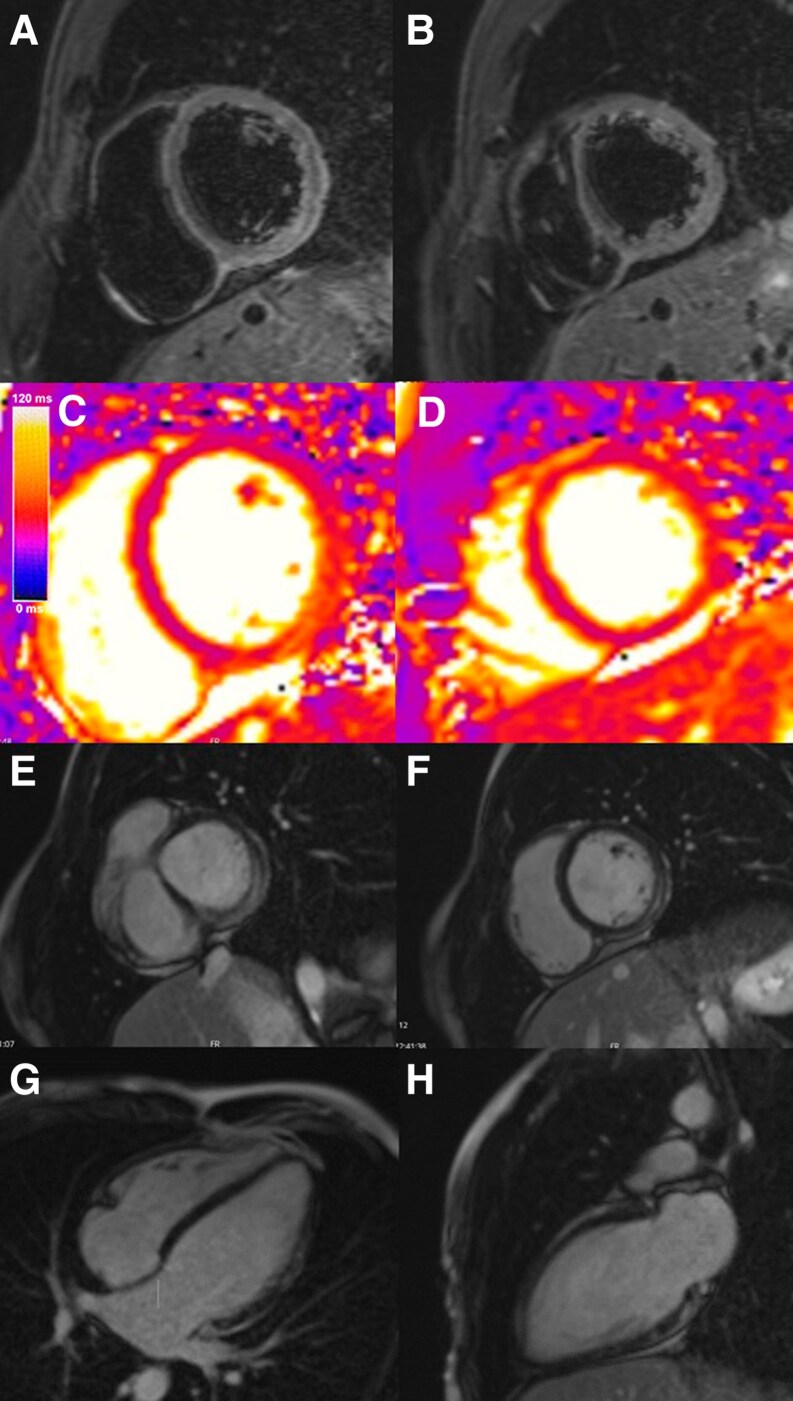
Patient 1 cardiac magnetic resonance T2 short-tau inversion recovery (STIR) (*A* and *B*), T2 mapping (*C* and *D*), late gadolinium enhancement pattern, (*E* and *F*) short axis, (*G*) four-chamber, and (*H*) two-chamber views.

A 23-year-old male with no significant personal or family medical history presented to our hospital with 3 days of fever, precordial chest pain that worsened with inspiration, and odynophagia. The patient’s examination was unremarkable. The electrocardiogram showed sinus tachycardia 110 b.p.m., slight PR interval, and ST-segment depression in v3–6 (*Figure 1*). The initial Troponin-T was elevated at 206.0 ng/mL (normal value < 18.0 ng/mL), reaching its peak on the third day of hospital admission at 560.4 ng/mL. Transthoracic echocardiogram showed normal left ventricle (LV) systolic function, no regional wall motion abnormalities (RWMA), and no pericardial effusion. Due to the combination of chest pain, elevated troponin levels, and non-specific ST-segment abnormalities, a cardiac CT scan was performed, given his young age and the low pre-test probability, ruling out coronary artery disease. To further investigate the suspected diagnosis of myocarditis, a cardiac magnetic resonance was performed, which revealed a dilated left ventricle (end-diastolic volume of 114 mL/m^2^, normal < 95 mL/m^2^) with mildly reduced left ventricle ejection fraction (LVEF) of 51%, no RWMA, and a non-dilated right ventricle with preserved systolic function. The T2 mapping value was increased at the level of the basal and mid-inferolateral wall (55 ms, normal < 52 ± 3 ms) suggestive of oedema, and there was extensive subepicardial late gadolinium enhancement (LGE) in the basal and mid-segments of the inferior, inferolateral, and anterolateral walls (*Figure 2*). These findings raised suspicion of a ‘hot phase’ of cardiomyopathy, prompting genetic testing.

He was started on a beta-blocker, an angiotensin-converting enzyme inhibitor, and colchicine due to pleuritic pain and suspected pericardial involvement, with progressive improvement of symptoms and normalization of troponin during the hospital stay. At follow-up, the genetic test result was positive for a ‘likely pathogenic’ mutation in the LMNA gene (MIM * 150330), c.490G > A, p.(Asp164Asn). He suspended colchicine and maintained the remaining medical therapy. Family members were referred for screening. At 7 months, he repeated cardiac magnetic resonance (CMR), which showed a mildly dilated LV with a normal LVEF of 56%, normal T2 mapping value, and reduction in LGE extension, with a remaining small focal zone of LGE present at the basal segment of the inferior wall. His calculated LMNA risk score for life-threatening arrhythmias (https://lmna-risk-vta.fr/) is 6.4%. He has maintained follow-up at the outpatient clinic with regular transthoracic echocardiogram, electrocardiogram (ECG), Holter, and exercise stress testing, without significant findings or adverse outcomes up to date.

### Patient 2

**Figure 3:**
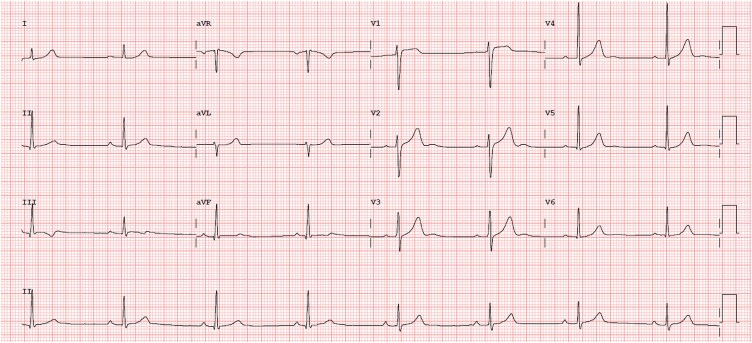
Patient 2 electrocardiogram showing sinus bradycardia, a first-degree AV block, and unspecific repolarization alterations with T wave inversion in DIII.

**Figure 4:**
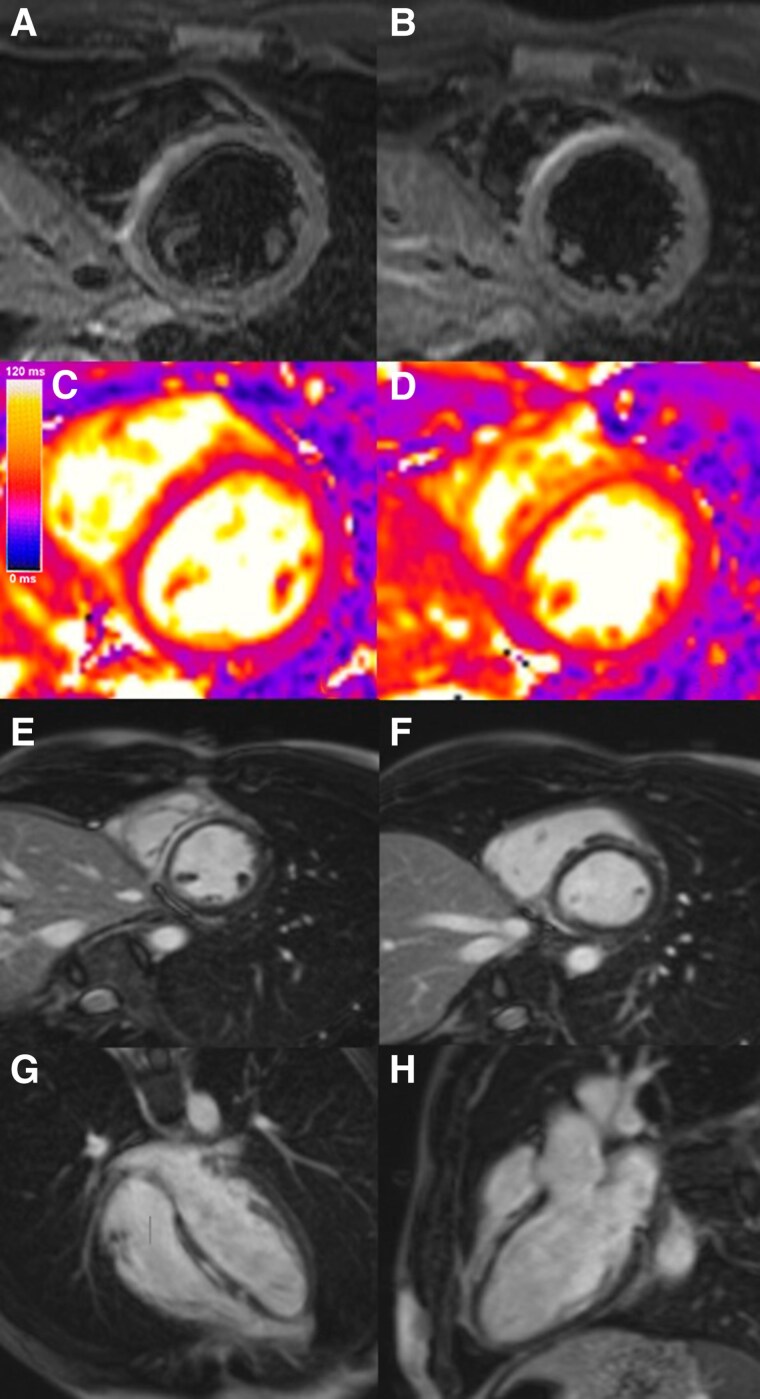
Patient 2 cardiac magnetic resonance T2 short-tau inversion recovery (STIR) (*A* and *B*), T2 mapping (*C* and *D*), late gadolinium enhancement pattern, (*E* and *F*) short axis, (*G*) four-chamber, and (*H*) three-chamber views.

A 26-year-old man without significant personal medical history presented to our hospital with 2 days of pleuritic chest pain. He mentioned a viral syndrome with fever and cough a week earlier. The cardiovascular exam was unremarkable. The ECG showed sinus bradycardia, a first-degree auriculoverntricular (AV) block and unspecific repolarization alterations with T wave inversion (TWI) in the DIII (*Figure 3*). High-sensitivity Troponin-T was elevated at the initial evaluation at 1342.0 ng/L, and it rose to a maximum of 2234.0 ng/L on the second day of the hospital stay. Transthoracic echocardiogram showed a non-dilated LV with normal systolic function, no RWMA, and no pericardial effusion. Given the marked rise in cardiac biomarkers and the repolarization abnormalities on ECG, an invasive coronary angiogram was performed to exclude obstructive coronary artery disease. Cardiac magnetic resonance was performed 6 days after hospital admission, and it revealed a non-dilated LV with LVEF of 64% and a normal-sized right ventricle (RV) with normal systolic function. The pre-contrast myocardial T1 relaxation time was slightly increased (1058 ms, normal < 1004 ± 24 ms), and the T2 mapping value was elevated at the level of the basal and mid-septum (56 ms, normal < 52 ± 3 ms), suggestive of oedema. There was linear mid-wall LGE on the basal and medial segments of the anterior and inferior septum (*Figure 4*). Regarding the patient’s family medical history, his mother had died at 54 years old of sudden cardiac death (SCD), and his father died at 58 years old of unknown causes. He had five siblings without known medical history, and the remaining family members were healthy.

Given the clinical and laboratory findings of myocardial injury, the familiar history of SCD, the sinus bradycardia and first-degree AV block, and the LGE pattern on CMR, the patient was referred to genetic testing, which was positive for a heterozygous pathogenic mutation in the DSP gene (MIM * 125647), c.495del, p.(Ser166Profs*30). After a multidisciplinary discussion with a focus on the arrhythmic risk, the patient was referred to implantation of subcutaneous implantable cardioverter defibrillator (ICD). He has maintained follow-up at our outpatient clinic with regular ETT, ECG, Holter, and exercise stress testing without significant findings, ICD events, or adverse outcomes up to date.

## Discussion

This case series highlights the clinical features and diagnostic challenges posed by the ‘hot phase’ of ACM, which is an increasingly recognized form of ACM presentation that occurs as a result of acute myocardial inflammatory injury, supported by laboratory and/or imaging findings. Although the pathophysiological mechanisms remain incompletely understood, it has been hypothesized that the disappearance of healthy myocardium, which constitutes the diagnostic hallmark of ACM, could be the consequence of an inflammatory injury with cell death, followed by fibrofatty repair, and thus an infectious or immune myocardial reaction could trigger a hyperinflammatory response. The findings of inflammatory cells infiltrate in endomyocardial biopsies and autopsy specimens of ACM further corroborate this theory.^1,3-5^

Both patients in this series are young males who presented acute onset of pleuritic chest pain, elevated troponin levels, and repolarization abnormalities on ECG, mimicking an acute cardiac inflammatory syndrome similar to viral myocarditis. It should be noted that current non-invasive diagnostic criteria for myocarditis, such as the Lake Louise criteria,^6^ when applied without histological confirmation, may lead to the misclassification of ‘hot phase’ ACM as myocarditis.

The overlapping features of these different diagnoses underscore the importance of utilizing advanced imaging modalities, particularly CMR, to improve diagnostic accuracy and arrhythmic risk stratification. Whereas in viral myocarditis, typical CMR findings include focal myocardial oedema with corresponding LGE often in a subepicardial pattern involving the basal to mid-inferolateral and inferior segments, in ACM the fibrofatty infiltration can be heterogeneous, and the LGE present different patterns and extension. Although an overlap in LGE patterns may be noted, small observational studies have demonstrated that in the ‘hot phase’ presentation of ACM, a third of patients have LV systolic dysfunction, the majority of patients present epicardial LV LGE, and around a fifth have RV LGE and RV systolic dysfunction.^2,3^ Genetic testing allowed for confirmation of the diagnosis of ACM while also providing information regarding prognosis and arrhythmic risk. Mutations in the LMNA and DSP genes are known causes of ACM, with the LV often being the predominantly affected ventricle. The LMNA encodes lamin A and C proteins, essential nuclear envelope components, while DSP encodes desmoplakin, a crucial protein for cell–cell adhesion in cardiac tissues. Both these mutations are associated with a high rate of conduction disturbances, malignant ventricular arrhythmias, systolic dysfunction, and progressive advanced heart failure. The DSP gene mutation has been recognized as one of the most associated with ‘hot phases’ of ACM.^3,7^ Acute myocardial inflammation in ACM seems to have an important role in the natural history of the disease, and it can precede the onset of histological and electrical abnormalities.^8^ Genetic testing in patients presenting with acute myocarditis-like syndromes has shown that a significant subset harbours pathogenic or likely pathogenic variants linked to hereditary cardiomyopathies. Studies have reported that up to 22% of patients initially diagnosed with myocarditis carry such mutations, with DSP, LMNA, and TTN being the most frequently implicated genes.^3^

For patients with LMNA variants, the arrhythmogenic risk is a significant concern. According to the 2022 ESC Guidelines for the management of patients with ventricular arrhythmias and sudden cardiac death,^9^ an LVEF threshold of 50% or an LMNA risk score of 10% or higher indicates a substantial risk of sudden cardiac death and should prompt consideration for ICD implantation for primary prevention. In the Patient 1 case, given the LMNA risk score of 6.4% and preserved LVEF above 50%, after multidisciplinary discussion, the risk was not deemed high enough to warrant ICD implantation at this time, aligning with guideline recommendations.

For DSP variants, the arrhythmogenic risk assessment takes into account multiple factors beyond the LVEF. The 2023 ESC Guidelines for the management of cardiomyopathies^10^ suggest that ICD implantation may be warranted even when LVEF is higher than 35% if additional risk factors are present. In the case of Patient 2, given the family history of SCD and the imaging findings, the decision for subcutaneous ICD implantation was made to mitigate the risk of fatal arrhythmic events.

Recognizing this acute inflammatory phase as a potential mimic of myocarditis is crucial for accurate diagnosis and personalized therapeutic strategies, including heart failure management and cardiac device implantation. A multidisciplinary approach involving cardiologists, geneticists, and pathologists is vital for accurate diagnosis and appropriate clinical management. Further research and larger case studies are warranted to elucidate the underlying mechanisms and refine diagnostic algorithms for optimal patient care.

## Lead author biography


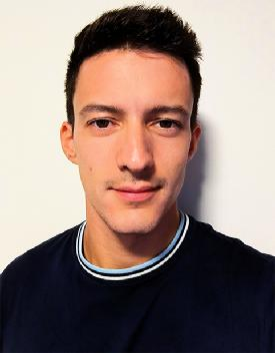


André Ferreira is a cardiology fifth year fellow at Santa Marta Hospital in Lisbon. He is interested in cardiac imaging and cardiomyopathies. He has other papers published as the first author and multiple abstracts in different journals.

## Data Availability

The data underlying this article will be shared upon reasonable request to the corresponding author.

## References

[1] Bariani R, Rigato I, Cipriani A, Marinas MB, Celeghin R, Basso C, et al Myocarditis-like episodes in patients with arrhythmogenic cardiomyopathy: a systematic review on the so-called hot-phase of the disease. Biomolecules 2022;12:1324. 36139162 10.3390/biom12091324 PMC9496041

[2] Todiere G, Barison A, Baritussio A, Cipriani A, Guaricci AI, Pica S, et al Acute clinical presentation of nonischemic cardiomyopathies: early detection by cardiovascular magnetic resonance. J Cardiovasc Med 2022; doi:10.2459/JCM.0000000000001412 36729634

[3] Monda E, Bakalakos A, Cannie D, O’Mahony C, Syrris P, Kaski JP, et al Prevalence of pathogenic variants in cardiomyopathy-associated genes in acute myocarditis. JACC Heart Fail 2024;12:1101–1111. 38573261 10.1016/j.jchf.2024.02.012

[4] Basso C, Thiene G, Corrado D, Angelini A, Nava A, Valente M. Arrhythmogenic right ventricular cardiomyopathy. Circulation 1996;94:983–991. 8790036 10.1161/01.cir.94.5.983

[5] Sen-Chowdhry S, Syrris P, McKenna WJ. Role of genetic analysis in the management of patients with arrhythmogenic right ventricular dysplasia/cardiomyopathy. J Am Coll Cardiol 2007;50:1813–1821. 17980246 10.1016/j.jacc.2007.08.008

[6] Ferreira VM, Schulz-Menger J, Holmvang G, Kramer CM, Carbone I, Sechtem U, et al Cardiovascular magnetic resonance in myocarditis: a JACC white paper. J Am Coll Cardiol 2018;72:3158–3176. 30545455 10.1016/j.jacc.2018.09.072

[7] Tiron C, Campuzano O, Fernández-Falgueras A, Alcalde M, Loma-Osorio P, Zamora E, et al Prevalence of pathogenic variants in cardiomyopathy-associated genes in myocarditis. Circ Genom Precis Med 2022;15:3. 10.1161/CIRCGEN.121.003408 35522179

[8] Bassetto G, Merlo M, Ferro MD, Setti M, Paldino A, Collesi C, et al Apoptosis, a useful marker in the management of hot-phase cardiomyopathy? Eur J Heart Fail 2024;26:590–597. 38414301 10.1002/ejhf.3173

[9] Zeppenfeld K, Tfelt-Hansen J, de Riva M, Winkel BG, Behr ER, Blom NA, et al 2022 ESC guidelines for the management of patients with ventricular arrhythmias and the prevention of sudden cardiac death: developed by the task force for the management of patients with ventricular arrhythmias and the prevention of sudden cardiac death of the European Society of Cardiology (ESC) endorsed by the Association for European Paediatric and Congenital Cardiology (AEPC). Eur Heart J 2022;43:3997–4126.36017572

[10] Arbelo E, Protonotarios A, Gimeno JR, Arbustini E, Barriales-Villa R, Basso C, et al 2023 ESC guidelines for the management of cardiomyopathies: developed by the task force on the management of cardiomyopathies of the European Society of Cardiology (ESC). Eur Heart J 2023;44:3503–3626. 37622657 10.1093/eurheartj/ehad194

